# Cancer of unknown primary eventually diagnosed as poorly differentiated prostate cancer: a case report

**DOI:** 10.1186/s13256-023-04118-2

**Published:** 2023-09-03

**Authors:** Kazutaka Iijima, Toshizo Takayama, Satoko Shindo, Rika Moku, Koya Sawai, Rio Honma, Naoki Hyakushima, Tomoshige Akino, Yumiko Oyamada, Yasushi Tsuji

**Affiliations:** 1 Department of Medical Oncology, Tonan Hospital, Sapporo, Hokkaido Japan; 2 Rumoi City Hospital Department of Gastroenterology, Rumoi, Hokkaido Japan; 3 Department of Otorhinolaryngology, Tonan Hospital, Sapporo, Japan; 4 Department of Urology, Tonan Hospital, Sapporo, Japan; 5 Department of Pathology, Tonan Hospital, Sapporo, Japan

**Keywords:** Cancer of unknown primary, Prostate cancer, Prostate-specific antigen, Positron emission tomography/computed tomography, Bone metastases, Cervical lymph node metastases

## Abstract

**Background:**

Prostate cancer has been well known to have a high prevalence among middle-aged and older men, with high incidence of metastases to the bone—the main metastatic site. However, prostate cancer among those less than 50 years of age is extremely rare, and neck swelling is seldom the initial symptom.

**Case presentation:**

We herein report case of a 47-year-old Japanese male with poorly differentiated prostate cancer that had been initially diagnosed as a cancer of unknown primary with multiple lymph node and bone metastases before reaching a definitive diagnosis. The patient has been started on endocrine therapy and is currently alive without progression.

**Discussion and conclusion:**

When locating the primary lesion in men with cancer of unknown primary, it is important to consider the possibility of prostate cancer, confirm serum prostate-specific antigen levels, and perform local prostate evaluation.

## Background

Prostate cancer (PrCa) is the second most common cancer and the fifth leading cause of death among men worldwide, with around 375,000 men dying each year [1]. PrCa has been well known to have a higher prevalence among middle-aged and older men. Moreover, incidence rates of PrCa have been found to increase steeply from age 50, with two-thirds of cases being diagnosed over the age of 70 [2].

PrCa most commonly metastasizes to bones (84%), distant lymph nodes (10.6%), liver (10.2%), and thorax (9.1%) [3]. In line with this fact, studies have shown that around 14.9–18% of patients diagnosed with PrCa already have distant metastases upon diagnosis [4, 5].

Thus, prostate evaluation is important when identifying the primary lesion of metastatic adenocarcinomas.

Here, we report a case of poorly differentiated PrCa that had been initially diagnosed as cancer of unknown primary (CUP) with multiple lymph node and bone metastases before reaching a definitive diagnosis.

## Case presentation

**Fig. 1:**
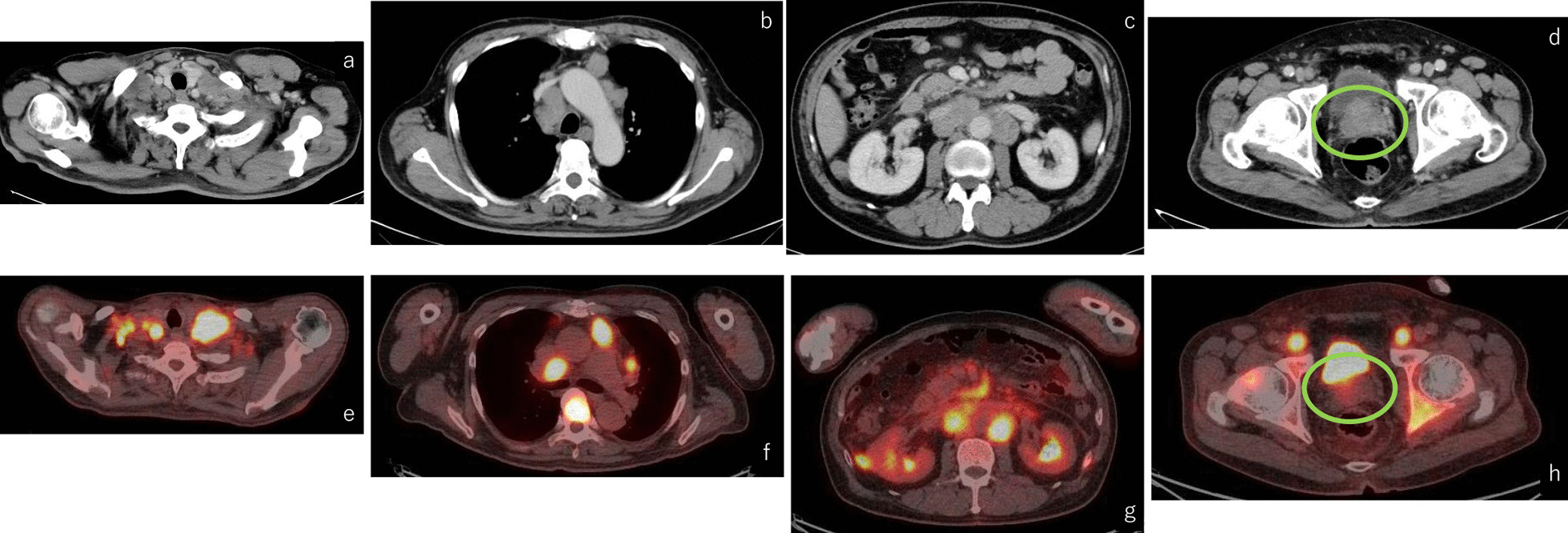
Computed tomography showing multiple swollen lymph nodes in both sides of his neck (**a**), mediastinum (**b**), abdomen (**c**), and pelvis (**d**). Positron emission tomography-computed tomography showing 18F-fluorodeoxyglucose accumulation in the same area and multiple bone metastases (**e**–**h**). Despite prostate enlargement, no FDG accumulation was observed (green circle)

A 47-year-old Japanese male with no significant past medical history presented in July 2021 complaining of neck swelling. After visiting his primary physician, the patient underwent computed tomography (CT), which showed multiple swollen lymph nodes in both sides of his neck, mediastinum, abdomen, and pelvis (Fig. 1a–d). He was admitted for further evaluation and management, and was referred to an otorhinolaryngologist. Subsequent cervical lymph node biopsy revealed poorly differentiated adenocarcinoma.

Thereafter, the patient underwent whole-body 18F-fluorodeoxyglucose (FDG) positron emission tomography-computed tomography (PET-CT), which revealed, in addition to the already identified lymph nodes, multiple FDG accumulations in the bones throughout the patient’s body (Fig. 1e–h).

However, given that the primary lesion could not be identified at the time, he was referred to our hospital in August with a diagnosis of CUP with multiple lymph node and bone metastases.

Physical examination showed multiple palpable lymph nodes in both sides of his neck. No other remarkable abnormalities had been found. He had no previous medical history and no family history of cancer including prostate cancer.

Initial laboratory tests showed elevated levels of lactate dehydrogenase (431 U/L), alkaline phosphatase (355 U/L), uric acid (7.2 mg/dL), C-reactive protein (3.3 mg/dL), blood sugar (190 mg/dL), and glycated hemoglobin (8.1%, NGSP) but decreased levels of albumin (3.4 g/dL).

We conducted a comprehensive evaluation for tumor markers and found elevated levels of carcinoembryonic antigen (8.6 ng/mL), carbohydrate antigen 19–9 (66 U/mL) and CA125 and remarkably high levels of prostate-specific antigen (PSA) (782 ng/mL).

Given that the patient was complaining of bone pain, radiation therapy (RT) for bone metastases [left ischium 20 Gy/4fr, thoracic spine (Th2–Th8) 30 Gy/10fr, right femur 25 Gy/5fr] was started. Irradiation and oral Meloxicam 10 mg/day improved his bone pain. For his diabetes, oral antidiabetics (Metformin 500 mg/day and Sitagliptin 50 mg/day) were started, resulting in good glucose control and glycated hemoglobin was normalized 3 months later.

**Fig. 2:**
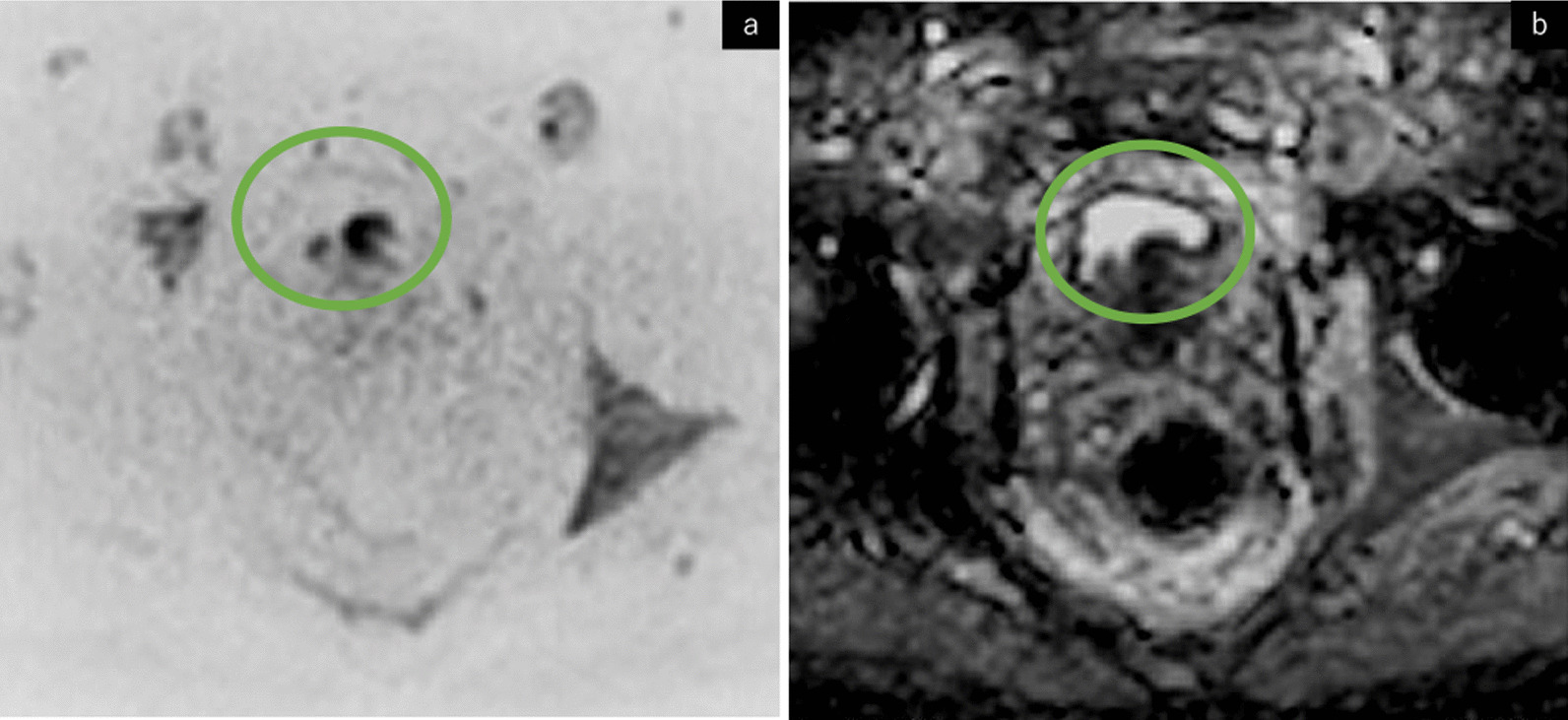
Magnetic resonance imaging showing the central to transitional area of the prostate protruding toward the bladder, and diffusion-weighted images (**a**) and apparent diffusion coefficient (**b**) mapping showing diffusion disorders (green circle)

The CT and PET/CT performed so far, as well as palpation of the prostate showed no obvious abnormalities. However, given that we suspected PrCa as the primary site based on the elevated PSA levels, prostate biopsy was performed in September 2021. Concurrently, magnetic resonance imaging (MRI) showed that the central to transitional area of the prostate protrudes toward the bladder, and diffusion-weighted images (DWI) and apparent diffusion coefficient (ADC) mapping showed diffusion disorders PI-RADS score 5 (Prostate Imaging Reporting and Data System) (Fig. 2).

**Fig. 3:**
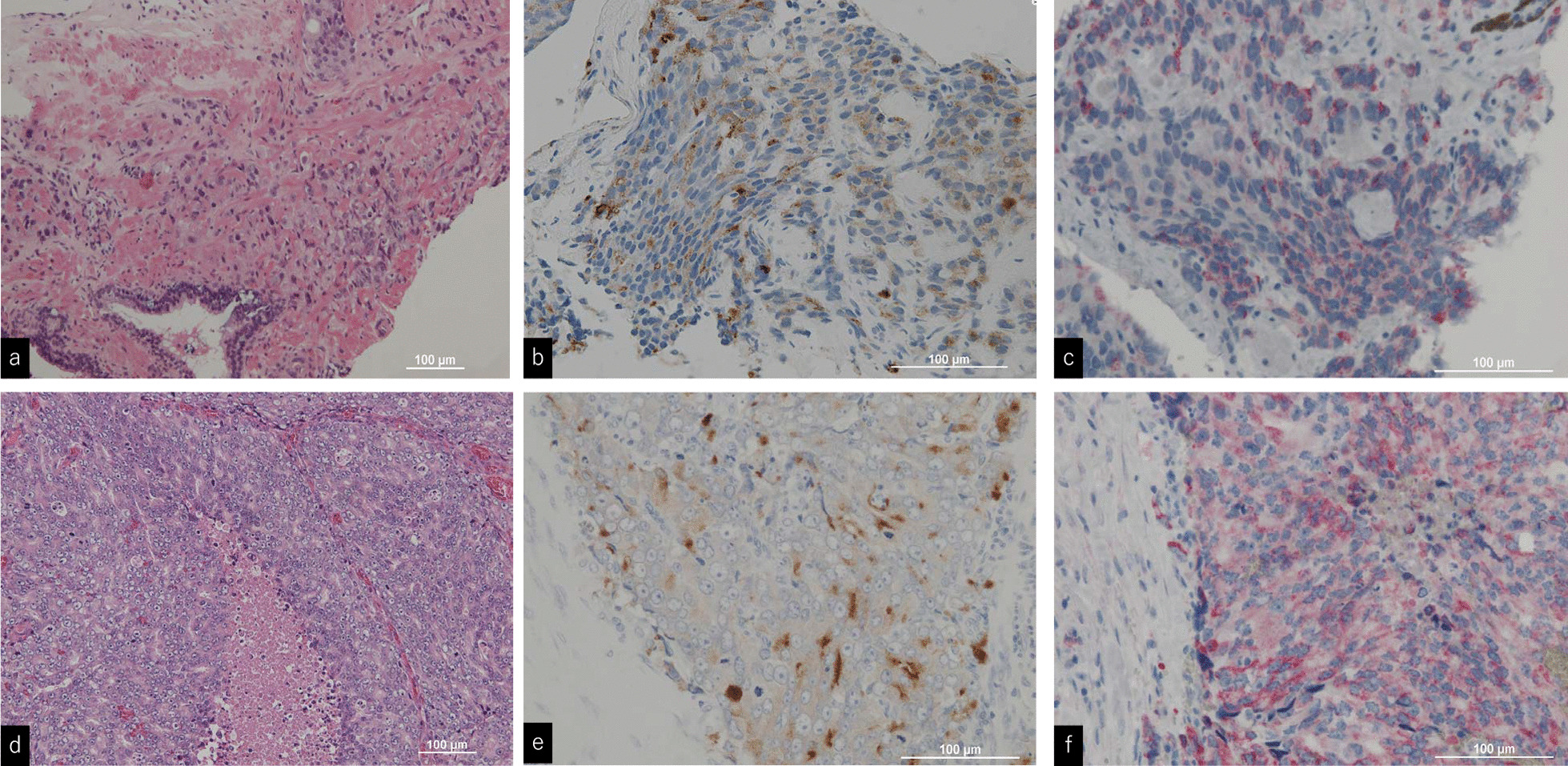
After Hematoxylin and Eosin (HE) staining of the prostate (**a**),and cervical lymph node (**d**), prostate biopsy revealed poorly differentiated adenocarcinoma (Gleason score 9–10). Prostate-specific antigen staining (**b**, **d**) and α-methylacyl coenzyme A racemase staining (**c**, **f**) of the prostate and cervical lymph node biopsy specimen were positive

Tissue obtained through biopsy revealed poorly differentiated ductal adenocarcinoma Gleason score 5 + 5. There were no other types of histological variants—apart of adenocarcinoma—in the prostate biopsy specimen. Immunohistochemical analysis was negative for pan-cytokeratin(CK) 7 and CK 20, positive for PSA and α-methylacyl coenzyme A racemase (AMACR) staining of the prostate and the cervical lymph node biopsy specimen (Fig. 3), and a definitive diagnosis of multiple lymph node metastases and bone metastases of PrCa (T1cN1M1b, stage IV; UICC 8th edition, 2017) was confirmed.

The patient was then started on androgen deprivation therapy (ADT: luteinizing hormone-releasing hormone (LH-RH) antagonist once every 4 weeks) plus abiraterone (1000 mg/day) and prednisone (5 mg/day).

**Fig. 4:**
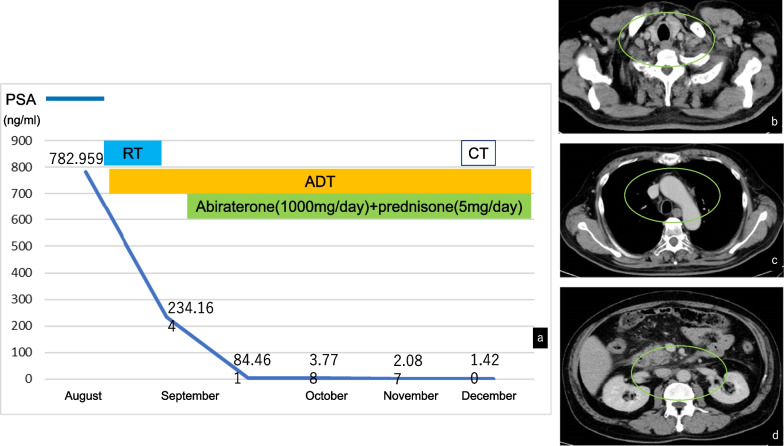
Clinical course of the present case and changes in prostate-specific antigen (PSA) levels since the start of the treatment (**a**). Prostate-specific antigen decreased, whereas computed tomography evaluation after the start of treatment (**b**–**d**) revealed a significant decrease in lymph node swelling (green circle)

Since treatment initiation, the patient’s PSA has decreased and eventually normalized, and systemic lymph node swelling has subsided significantly (Fig. 4). The patient has had no complaints of pain related to bone metastases, and his treatment can continue even half a year later.

## Discussion

Despite improvements in diagnostic techniques, CUP has been estimated to account for approximately 3–5% of new cancer diagnoses worldwide [6]. Although most patients with CUP have a poor response to treatment and have poor prognosis, a fraction does have a favorable prognosis due to potential good response to treatment, for example, male patients with only bone metastases and increased serum PSA [6–8]. If the primary lesion can be identified, cancer-specific treatment is recommended, although such cases are excluded from the strict definition of CUP [7].

Whole-body CT is generally utilized to identify the primary lesion in CUP and has been recommended by various guidelines [6, 7]. Although no evidence has yet suggested the utility of FDG-PET/CT in locating the primary lesion of CUP, this modality is often useful, particularly in head and neck cancers [9]. However, studies have pointed out that PrCa is difficult to detect by CT given the low FDG accumulation such as in our case [10, 11]. Therefore, overconfidence in CT and FDG-PET/CT findings and the ensuing convenient diagnosis of CUP may end up being harmful to the patient.

In European countries and the United States, nuclides such as choline (11C-choline, 18F-fluorocholine), which is poorly excreted in urine, have been used for the diagnosis of prostate cancer. 18F-FDG accumulates better in malignant tumors with enhanced glucose metabolism, whereas choline accumulates more in malignant tumors with active phospholipid synthesis in their cell membrane, such as PrCa [12, 13]. While choline PET/CT can be useful for diagnosing prostate cancer, one drawback is that it can only be performed by a limited number of specialized research institutes [13].

MRI has been considered an objective and reliable diagnostic imaging method for the local staging of PrCa, despite its unsuitability for screening [14]. Tumor marker assay has also been useful in the identification of the primary lesion of CUP, being recognized especially for its utility in identifying individuals with good prognosis [7], including those with PrCa and likely to achieve prolonged survival due to good response to treatment. Moreover, serum PSA measurements have been recommended for middle-aged and elderly men with bone metastases of unknown primary adenocarcinoma. Estimates have suggested that 96–100% of patients with stage IV PrCa have increased serum PSA levels, while a prospective study on serum PSA levels showed that a primary lesion was found in 50–80% of CUP patients with serum PSA > 10 ng/mL, and distant metastases were observed among those with serum PSA > 100 ng/mL [15–17].

The presence of lymph node metastases in addition to bone metastases has added a level of complexity to our case. Given its lesser invasiveness and good utility, serum PSA assay is meaningful as a routine examination for middle-aged and elderly men with CUP. However, it has been reported that in 50–80% of patients PrCa was not detected when performing prostate biopsy based only on high serum PSA levels [18], suggesting the need for a systematic evaluation of not only tumor markers but also imaging and pathological findings on biopsy specimens from metastatic lesions.

Immunohistochemical staining (IHC) plays a very important role in the workup of adenocarcinoma of unknown primary, and PSA is the most widely known biomarker in the diagnosis of prostate cancer. In addition, AMACR has high sensitivity and specificity in prostate cancer, and overexpression of AMACR in prostate cells is associated with poor patient prognosis. In this case, both the prostate and cervical lymph node biopsies showed positive staining results for PSA and AMACR, leading to the diagnosis of cervical lymph node metastasis of prostate cancer [19, 20].

Reports have shown that ADT plus abiraterone and prednisone leads to better overall survival (OS) compared to ADT monotherapy as a treatment for hormone-sensitive metastatic PrCa [21]. In addition, recent studies have suggested that combination therapy with ADT, docetaxel, and abiraterone (+ prednisone) prolongs progression-free survival in hormone-sensitive PrCa with extensive metastases [22]. Although this case had a high tumor volume upon diagnosis, hormone therapy was administered at the patient's request considering the adverse events associated with cytotoxic agents, and it achieved marked tumor shrinkage. If tumor control becomes difficult in the future, we are ready to reconsider chemotherapy.

## Conclusion

We experienced a case of CUP eventually diagnosed as poorly differentiated PrCa. The patient had other than bone metastases and no urinary tract symptoms. When identifying the primary lesion in men with CUP, considering the possibility of PrCa, confirming serum PSA levels, and performing local evaluation of the prostate are crucial elements of the diagnostic approach.

## Data Availability

Our data included personal patient data. Additional data are available from the corresponding author upon reasonable request.

## Abbreviations

ADC: Apparent diffusion coefficient
ADT: Androgen deprivation therapy
CA19-9: Carbohydrate antigen 19–9
CA125: Carbohydrate antigen 125
CEA: Carcinoembryonic antigen
CT: Computed tomography
CUP: Cancers of unknown primary
DWI: Diffusion-weighted images
FDG: 18F-2-fluoro-2-deoxy-d-glucose
HE: Hematoxylin eosin
LH-RH: Luteinizing hormone-releasing hormone
MRI: Magnetic resonance imaging
OS: Overall survival
PrCa: Prostate cancer
PET-CT: Positron emission tomography-computed tomography
PSA: Prostate-specific antigen
